# Gender differences in metabolic risk factor prevalence in a South African student population

**Published:** 2009-05

**Authors:** Carine Smith, M Faadiel Essop

**Affiliations:** Department of Physiological Sciences, Stellenbosch University, Stellenbosch, South Africa; Department of Physiological Sciences, Stellenbosch University, Stellenbosch, South Africa

## Abstract

**Summary:**

We determined selected risk factors for the metabolic syndrome and assessed the metabolic risk status (using IDF criteria) of third-year physiology students at Stellenbosch University (88 males and 178 females). Outcome measures included anthropometry [body mass index (BMI), waist circumference, waist-to-hip ratio], blood pressure (BP), resting pulse rate, and fasting blood glucose, total cholesterol and triglyceride levels. In addition, students completed a lifestyle questionnaire.

A number of gender-based differences were found, with male students displaying a greater incidence of risk factors for the metabolic syndrome: 6% of males versus 3% of females displayed a cluster of three risk factors. Twenty-five per cent of female students (but only 14% of males) exhibited waist circumferences above the accepted range, which was positively correlated, for males and females, with both systolic and diastolic BP, and in females only, also with total cholesterol levels. Male students on average exercised more than their female counterparts, but also exhibited poorer eating habits. Average blood triglyceride levels for both male and female students exceeded the accepted threshold (1.85 ± 1.62 mmol/l and 2.15 ± 1.79 mmol/l, respectively).

We concluded that metabolic risk factors were evident in a much younger population than commonly expected. Moreover, the gender-specific differences observed may impact on future risk assessment and preventative measures adopted.

## Summary

A World Health Organisation report warns of the escalating global burden of cardiovascular disease (CVD), projecting that it will become the major cause of death and disability worldwide by 2020.^1^ In agreement, a recent study investigating the prevalence of CVD in South Africa, Brazil, India, Russia, Portugal and the USA projects a marked increase for the incidence of CVD in developing countries.^2^ Although its underlying causes are multiple and its progression may take several years, researchers have suggested that the marked surge in CVD rates in developing countries may be due to accelerated urbanisation and associated lifestyle changes (e.g. increased fat consumption, obesity and decreased physical activity).^3^ The higher mortalities and morbidities associated with increased CVD rates will have serious socio-economic implications, including disruption of family units, greater healthcare costs and diminished productivity.

An emerging paradigm suggests that a cluster of metabolic abnormalities, referred to as the metabolic syndrome, is associated with increased risk for both the development of type 2 diabetes and CVD.^4^,^5^ Here the underlying rationale was to provide a clinically accessible diagnostic tool that will allow for the early identification of individuals at risk for the development of type 2 diabetes and/or CVD. In broad terms, the metabolic syndrome is characterised by a ‘deadly quartet’, including impaired glucose regulation, dyslipidaemia, hypertension and obesity.^4^,^5^ However, some of these parameters offer relatively limited information and therefore require a certain degree of re-evaluation. For example, obesity is typically assessed by employing the body mass index (BMI) despite it not providing information regarding the actual site(s) of excess fat deposition in the body. Since abdominal fat deposition is associated with more serious health implications than fat accumulation elsewhere,^3^ the International Diabetes Federation (IDF) now includes increased waist circumference as a pre-requisite for the diagnosis of the metabolic syndrome.^4^,^5^

Recent epidemiological data indicate that metabolic abnormalities representative of the metabolic syndrome may manifest at an earlier age than previously seen.^4^,^6^-^9^ In light of this, we tested the hypothesis that metabolic syndrome risk factors are high in an undergraduate South African university student group, assessing several parameters, such as anthropometry, blood pressure, resting pulse rate, as well as selected blood parameters.

## Methods

*Subject recruitment*: 266 student volunteers (88 males, 178 females) were recruited during student practical sessions held in the Department of Physiological Sciences at Stellenbosch University (South Africa). All students were verbally informed regarding the aims of the study, and were also given a chance, at the end of the practical session, to anonymously volunteer their results (the assessments formed part of a formal practical session) for use in this study. This study was ethically approved by the Stellenbosch University Subcommittee C Research Ethics Committee, and was conducted according to the Helsinki Declaration of 1975 (as revised in 1983).

*Data collection*: For logistical reasons we were only able to assess four of the five risk factors for the metabolic syndrome according to IDF criteria, i.e. blood pressure, abdominal obesity, and fasting blood glucose and triglyceride levels. Total cholesterol was included, but high-density lipoprotein (HDL) fractions were not determined.

Anthropometric assessments were performed by experienced individuals in accordance with ISAK (International Society for the Advancement of Kinanthropometry) standards. Body mass (kg) was recorded to the nearest 0.5 kg (Precision Health Scale, A&D Company, Japan) and height (m) was measured to the nearest 0.5 cm using the stretch plane method, with the head placed in the Frankfort plane, and a stadiometer (Invicta, IP 1465, UK). From these measurements, body mass index (BMI) was calculated (BMI = kg/m^2^). Waist and hip circumferences were measured (students in upright position) at the narrowest girth in the waist area and at the level of the greatest posterior protuberance of the buttocks, respectively, using a flexible steel measuring tape (W606PM, Lufkin, Canada).

Resting blood pressure and pulse rates were determined in subjects at rest, i.e. sitting down for at least three minutes and using an automated sphygmomanometer (BP3BA0, Microlife AG, Switzerland). Two independent readings were performed for each subject. Fasting whole blood triglyceride, glucose and total cholesterol concentrations were measured immediately after obtaining capillary blood by finger pricking (Accutrend GCT system, Roche, Switzerland). Of all students assessed, 2.6% (*n* = 7) indicated that they were on chronic medication, with only one student medicated specifically for high blood pressure.

We used IDF criteria to assess the metabolic syndrome risk factors: blood pressure ≥ 130/85 mmHg, fasting glucose levels ≥ 5.6 mmol/l, fasting triglyceride levels ≥ 1.69 mmol/l and waist circumference ≥ 94 cm (males) and ≥ 80 cm (females). Data regarding ethnicity, parental education level, family history of CVD and/or type 2 diabetes, as well as dietary habits (e.g. habitual consumption of caffeine, salt and fast food), were recorded using a simple questionnaire designed for this purpose.

*Data analysis:* Descriptive statistical analysis was performed using Excel (MS Office). Gender differences were established by one-way ANOVA after normal distribution of data had been established, and relationships between different parameters were assessed by calculation of Pearson’s correlation coefficients (Statistica v.7, StatSoft Software). A *p*-value of 0.05 was set as the level of significance. All results are presented as means ± standard deviations, unless otherwise stated.

## Results

Overall, anthropometric measures were within the expected ranges for both genders (Table 1). However, we found that 25% of female students exhibited waist circumferences above the IDF-accepted cut-off point of 80 cm. Conversely, 14% of male students exhibited a waist circumference above the IDF threshold of 94 cm. On average, both fasting glucose and total cholesterol levels were within the normal ranges (and below the threshold for metabolic syndrome risk) for both genders (Table 1). However, average fasting triglyceride levels were above the threshold in both gender groups (Table 1), with 35% of male and 37% of female individuals exhibiting high fasting triglyceride levels. In addition, in males, average blood pressures (systolic and/or diastolic) exceeded cut-off points in 44% of subjects, while 20% of females had increased systolic or diastolic blood pressures.

**Table 1:** Summary Of Anthropometric And Physiological Parameters Assessed In Undergraduate Students

	*Male (n = 88)*	*Female (n = 178)*
Age (year)	22 ± 1	21 ± 1
Height (cm)	178 ± 8	168 ± 7*
Body mass (kg)	78 ± 15	62 ± 10*
Body mass index (BMI)	24.7 ± 4.3	22.10 ± 3.10*
Waist circumference (cm)	83.3 ± 10.8	75.3 ± 9.1*
Hip circumference (cm)	94.0 ± 10.2	93.2 ± 9.10
Waist/hip ratio	0.89 ± 0.08	0.81 ± 0.08*
Systolic blood pressure (mmHg)	129 ± 12	116 ± 12*
Diastolic blood pressure (mmHg)	82 ± 12	76 ± 10*
Resting pulse rate (beats/min)	68 ± 12	72 ± 13
Total cholesterol levels (mmol/l)	4.4 ± 0.6	4.3 ± 0.9
Glucose levels (mmol/l)	4.1 ± 1.1	4.1 ± 1.0
Triglyceride levels (mmol/l)	1.85 ± 1.62	2.15 ± 1.79

*Significant differences between genders (*p* < 0.001).

**Fig. 1.**
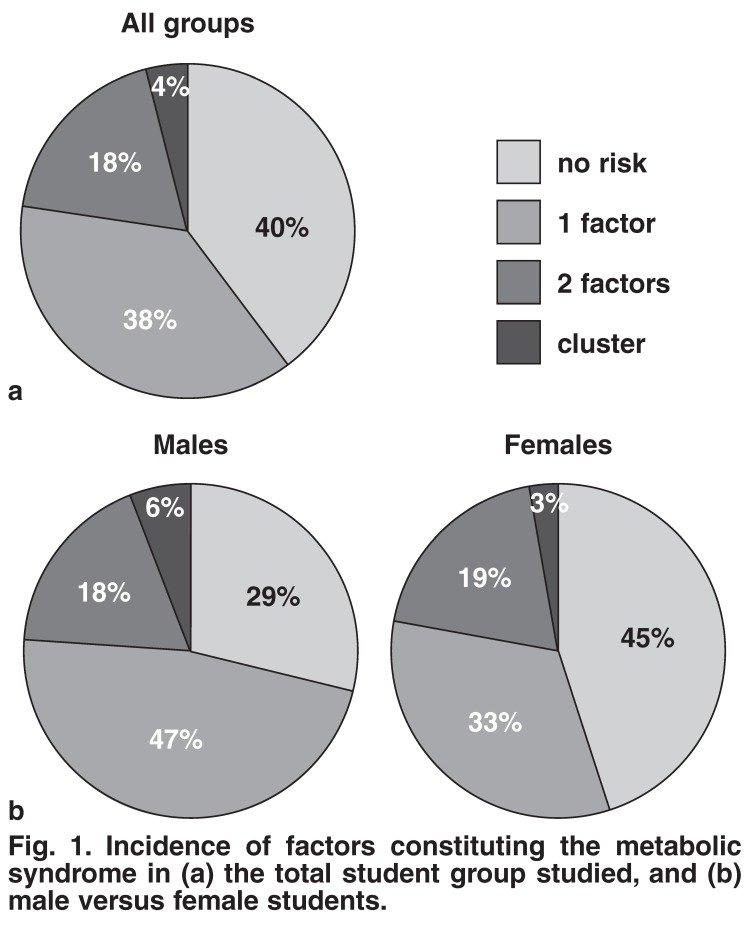
Incidence of factors constituting the metabolic syndrome in (a) the total student group studied, and (b) male versus female students.

When examining clustering of the risk factors measured, we found that only 40% of the total student population exhibited complete lack of any of the risk factors assessed (Fig. 1a). We also found that females displayed a higher percentage of zero risk factors versus males, (45 vs 29%) (Fig. 1b). Furthermore, 18 and 6% of males and 19 and 3% of females exhibited two and three metabolic syndrome risk factors, respectively (Fig. 1b). Overall, 4% of the student population presented with a cluster of three risk factors (Fig. 1a).

To gain further insight into these findings, we determined possible relationships between several of the parameters tested. As before, gender-based differences were evident. For both gender groups, the waist circumference correlated positively with both systolic (*p* < 0.001 and *p* < 0.0001 for males and females, respectively) and diastolic blood pressures (*p* < 0.001 and *p* < 0.0001 for males and females, respectively), and in the female group only, also with total cholesterol levels (*p* < 0.05).

By employing a purpose-designed questionnaire, additional insight was gained regarding the active lifestyle of students. Although males habitually performed significantly more physical exercise than females (5.8 ± 4.5 vs 3.6 ± 2.8 hours per week, *p* < 0.0001), they also on average consumed larger quantities of fast food (2.0 ± 1.6 vs 1.2 ± 1.1 units per week, *p* < 0.0001) and alcohol (4.9 ± 7.5 vs 2.4 ± 3.3 units per week, *p* < 0.001) compared to females.

To establish links between the risk factors exhibited and possible lifestyle-related or genetic factors, we investigated in more detail the gender subgroups with increased blood pressure (Table 2), and increased triglyceride levels (Table 3). For the high blood pressure subgroup, lifestyle and genetic factors included in our assessment did not present a clear predictor of elevated blood pressure. However, the gender difference regarding habitual exercise was once again evident (*p* < 0.01), as was the higher intake of alcohol in the male group (*p* < 0.01).

**Table 2:** Summary Of Hereditary And Lifestyle-Related Parameters In The Subgroup Of Students With Elevated Blood Pressure (BP)

	*Males*	*Females*
Total number in group	39 (44%)	36 (20%)
Systolic BP (mmHg)	137 ± 10	133 ± 8
Diastolic BP (mmHg)	89 ± 10	90 ± 10
Ethnicity (Caucasion : mixed ancestry : black)	32:6:1	28:3:5
Waist circumference (cm)	88 ± 10	81 ± 11
Number of smokers	11	4
Cigarettes per day in smoker subgroup	8.3 ± 5.6 (range 1–18)	11.4 ± 6.5 (range 5–20)
Number with family history of CVD	16	19
Number with family history of T2DM	15	15
Number with family history of CVD *and* T2DM	8	12
Caffeine consumption (units/day)	2 ± 2 (range 0–10)	2 ± 2 (range 0–8)
Habitual use of salt	Low to moderate	Low to moderate
Fast food consumption (meals/week)	1.8 ± 1.6 (range 0–9)	1.4 ± 1.2 (range 0–5)
Alcohol consumption (units/week)	6.1 ± 9.3	1.7 ± 2.2
Habitual exercise (hours/week)	6.6 ± 5.0 (range 0–24.5)	4.1 ± 3.0 (range 0–13)
Blood triglyceride level (mmol/l)	2.27 ± 1.43	2.17 ± 1.18
Number presenting with increased TG	15 (38%)	13 (36%)

BP = blood pressure; TG = blood triglyceride levels; CVD = cardiovascular diseases; T2DM = type 2 diabetes mellitus.

For the high triglycerides subgroup, the gender difference with regard to habitual exercise was not significant (*p* = 0.36). However, males in this group again consumed significantly more alcohol (*p* < 0.05), and also fast food (*p* < 0.001), compared to females. No direct correlation between habitual exercise and blood pressure or triglyceride level was evident in any gender group or subgroup. Interestingly, increased blood pressure appears to be a predictor of high blood triglycerides in the population studied, with just under 40% of subjects of both genders with high blood pressure also exhibiting high triglyceride levels (Table 2). However, the same was not true when considering high triglyceride levels as a predictor of blood pressure. While almost 50% of males with high triglyceride levels also exhibited high blood pressure, this was present in only 7% of females with high triglyceride levels.

**Table 3:** Summary Of Potential Contributing Factors In The Subgroup Of Students With Elevated Blood Triglyceride Levels

	*Males*	*Females*
Total number in group	31 (35%)	66 (37%)
Systolic BP (mmHg)	129 ± 12	117 ± 12
Diastolic BP (mmHg)	84 ± 11	77 ± 11
Ethnicity (Caucasion : mixed ancestry : black)	23:6:2	58:5:3
Waist circumference (cm)	86 ± 13	76 ± 10
Number of smokers	8	5
Cigarettes per day in smoker subgroup	8.7 ± 7.8 (range 1–25)	8.4 ± 7.1 (range 2–20)
Number with family history of CVD	12	18
Number with family history of T2DM	12	19
Number with family history of CVD *and* T2DM	5	9
Caffeine consumption (units/day)	2 ± 2 (range 0–7)	2 ± 2 (range 0–6)
Habitual use of salt	Low to moderate	Low to moderate
Fast food consumption (meals/week)	2.2 ± 1.7 (range 0–7)	1.2 ± 0.9 (range 0–4)
Alcohol consumption (units/week)	6.1 ± 9.3	2.7 ± 3.2
Habitual exercise (hours/week)	4.7 ± 4.0 (range 0–12)	4.0 ± 2.9 (range 0–15)
Blood triglyceride level (mmol/l)	3.08 ± 1.47	3.26 ± 1.21
Number presenting with increased TG	15 (48%)	12 (7%)

BP = blood pressure; TG = blood triglyceride level; CVD = cardiovascular diseases; T2DM = type 2 diabetes mellitus.

## Discussion

Recent studies show that metabolic risk factors such as these for the metabolic syndrome may be manifesting at younger ages than previously seen.^4^,^6^-^9^ In light of this, we hypothesised that metabolic syndrome risk factors would already be present in undergraduate university students. We investigated our hypothesis by assessing third-year physiology students based at Stellenbosch University. The main findings of this study are: (1) a relatively high incidence of the metabolic syndrome risk factors in a young student population, and (2) striking gender-based differences, with relatively more females displaying increased waist circumferences, while more males exhibited increased blood pressure.

We found that 4% of the students presented with a cluster of three or more risk factors. Furthermore, 18% of the total student population investigated presented with two risk factors for the metabolic syndrome. Since only four of the five IDF-defined risk factors were assessed in the current study, we propose it is likely that these figures may be higher. Similar studies conducted in other developing countries (Brazil and Turkey) reported a slightly lower prevalence of two to three metabolic risk factors (14–21%) in populations of schoolchildren.^6^,^7^

We propose that the socio-economic status and the degree of urbanisation of students attending Stellenbosch University may be key factors underlying the relatively high incidence of risk factors of the metabolic syndrome in the student population investigated. The lifestyle questionnaire indicated that students generally came from homes where parents (on average) had completed at least one course/degree at a tertiary institution. This can probably be linked to higher socio-economic status. An association was previously found between the level of parental education and resultant socio-economic status and the incidence of metabolic risk factors in younger individuals.^6^,^9^ Moreover, this trend was more significant in developing^6^,^7^ than developed countries.^8^

We further propose that the degree of urbanisation may also play a role in the higher incidences of risk factors of the metabolic syndrome in the student population studied, since this has been previously shown in other populations.^10^,^11^ Our study population largely consisted of urbanised individuals who were more exposed to the negative influences associated with modern-day urban dwelling, for example, fast-food eateries, greater stress and lack of physical exercise.

When the data were analysed in a gender-dependent manner, the males displayed a higher incidence of clustering of risk factors for the metabolic syndrome compared to females (6 vs 3%). The increased blood pressures observed in the male students were likely to be associated with lifestyle choices and/ or family history of related diseases. Indeed, in the subgroup of students with high blood pressure, a relatively high percentage had a family history of either cardiovascular disease or type 2 diabetes, or both. Furthermore, male students (from both subgroups studied) exhibited poor lifestyle choices, such as increased habitual fast food and/or alcohol intake relative to their female peers (Tables 2 and 3). This is in accordance with a recent study reporting cumulative risk for high blood pressure in the presence of poor lifestyle choices and a family history of cardiovascular disease.^9^

Although smokers generally exhibit higher blood pressure than non-smokers,^12^ only 13% of the total subject group (10% of females and 19% of males) admitted to cigarette smoking. Since only 40% of this subgroup was smoking 10 or more cigarettes per day, no comparison was made between smokers and non-smokers. From these data it appears less likely that smoking played an important role in higher incidences of blood pressure in the population group studied.

We propose that the higher intake of fast foods in male students may indeed have played a key role. An interesting future study would be to determine whether increased intake of fast foods in young males compromises arterial elasticity at this age.

Although the female students exhibited a lower incidence of risk factors compared to the males, a worrying factor was the high percentage that displayed increased waist circumference measurements. Waist circumference in the female study population was directly associated with systolic/diastolic blood pressures and cholesterol levels. Central obesity is thought to be the single most important risk factor contributing to the development of the metabolic syndrome. For example, it was suggested that it may be the one parameter that distinguishes between the manifestation of sub-clinical and clinical metabolic syndrome.^11^ Adipose tissue is now recognised as an active metabolic organ, secreting hormones and cytokines that may have both paracrine and endocrine effects on different tissue types.^13^ Furthermore, epidemiological data link it to increased risk for diabetes (especially in women)^14^ and mortality.^15^ Our finding that so many young female students exhibited waist circumferences exceeding the normal range is alarming since it is traditionally linked to older populations.^16^

The combination of high triglyceride levels and increased waist circumference is also a robust predictor of accelerated atherogenesis and related mortality in postmenopausal women.^17^ Indeed, our finding of a correlation between waist circumference and total cholesterol in the females studied supports this notion. It is not entirely clear why there was a higher incidence of increased waist circumference in the female students, since they appeared to have a lower intake of fast-food meals and alcohol than the males. We speculate that the female students were more sedentary and may have performed less exercise than males. Our lifestyle questionnaire and the fact that the females on average had higher resting pulse rates when compared to the average for males (72 ± 13 vs 68 ± 12 beats per minute; *p* < 0.05), supports this proposal.

We are of the opinion that our data, particularly for the male students, highlight the benefits of regular exercise and its importance in prevention of diseases associated with poor lifestyle choices. Moderate exercise is known to be an important role player in the prevention and treatment of hypertension. Recently, an exercise regimen of more than 30 minutes per day was recommended as an efficient treatment for elevated blood pressure.^18^ Almost 70% of the males in this study population sufficiently met this guideline. Moreover, even in the high blood pressure and triglyceride subgroups, the average exercise routine exceeded the minimum recommendation. This may in part explain why these males were not exhibiting more advanced symptoms of the metabolic syndrome despite their poor lifestyle choices, particularly dietary intake. Conversely, only 46% of the females met the requirements for adequate exercise. However, for the female subgroups (high blood pressure and triglycerides) the average hours of exercise exceeded the minimum recommendation.

Recent studies on younger children and adolescents have demonstrated that the degree of fatness (BMI) and fitness are important predictors of increased prevalence of risk factors of the metabolic syndrome, for example higher blood pressure and triglycerides.^19^,^20^ Although few individuals in this study would have been classified as obese, a relatively large number displayed increased waist circumference, elevated triglyceride levels, or low fitness levels, or a combination of these. Clinicians should therefore be made aware that individuals presenting with risk factors for cardiovascular disease or the metabolic syndrome do not always manifest this in their physical appearance. Furthermore, it is important to raise awareness among younger population groups regarding suitable lifestyle choices (exercise and dietary choices) and their potential benefit to delay the onset of future chronic diseases.

We acknowledge a few limitations to the current study. The student group that participated in this study may not be representative of the broader age-matched population in South Africa in terms of ethnicity and socio-economic status. In addition, the student group consisted of more females than males. We were also unable to assess high-density lipoprotein cholesterol levels due to logistical considerations. Future studies including this parameter and other relevant biomarkers linked to the metabolic syndrome, e.g. interleukin (IL)-6, tumour necrosis factor (TNF)-α and C-reactive protein (using high-sensitivity assays), should assist in further interpretation of the data obtained.

## Conclusion

We investigated the prevalence of a cluster of metabolic syndrome risk factors in a South African student population and found that these factors presented at a much younger age than commonly expected. Our data show some gender-based differences, with females displaying a higher incidence of increased waist circumference, whereas more males exhibited increased blood pressure. We propose that these differences were related to student behavioural patterns, such as the male students displaying poorer lifestyle choices, as seen in sub-optimal dietary habits. Such differences may impact on future risk assessment and preventative measures adopted, and we suggest that these parameters should be investigated in a broader student population. We also propose that it is imperative to screen young students in order to identify risk profiles for the metabolic syndrome relatively early on and thereafter initiate appropriate lifestyle changes.
